# Aktuelle Möglichkeiten und Herausforderungen bei der Therapie des laryngopharyngealen Refluxes

**DOI:** 10.1007/s00106-023-01280-3

**Published:** 2023-02-16

**Authors:** Daniel Runggaldier, Bram van Schie, Silvan Marti, Jörg E. Bohlender

**Affiliations:** 1 Klinik für Otorhinolaryngologie, Head and Neck Surgery, Abt. für Phoniatrie und klinische Logopädie, Universitätsspital Zürich, Frauenklinikstrasse 24, 8091 Zürich, Schweiz; 2 Universität Zürich, Rämistrasse 71, 8006 Zürich, Schweiz

**Keywords:** Laryngopharyngealer Reflux, Protonenpumpeninhibitoren, Alginat, Mediterrane Diät, Refluxogenic Diet Score, Laryngopharyngeal reflux, Proton pump inhibitors, Alginate, Mediterranean diet, Refluxogenic diet score

## Abstract

Der laryngopharyngeale Reflux (LPR) ist durch ein Aufstoßen von gastralem bzw. gastroduodenalem Sekret wie auch von gasförmigem Inhalt in den oberen aerodigestiven Trakt charakterisiert, wodurch eine Schädigung der Schleimhäute u. a. im Larynx- und Pharynxbereich resultieren kann. Eine Reihe von Symptomen wie das klassische retrosternale Brennen und saure Aufstoßen oder weitere, teils sehr unspezifische Symptome wie Heiserkeit, chronischer Husten, zervikales Globusgefühl oder Hypersekretion von Mukus können daraus resultieren. Bei heterogener Studienlage und dem Fehlen eines Goldstandards ist, wie kürzlich zusammengefasst, die Diagnosestellung des LPR schwierig und umstritten. Die Therapieoptionen des LPR werden aufgrund der dünnen Datenlage ebenfalls kontrovers diskutiert und können neben medikamentösen auch konservative diätetische Maßnahmen umfassen. Das Ziel der nachfolgenden Übersichtsarbeit ist daher, die verfügbaren therapeutischen Optionen des LPR kritisch zu diskutieren und für den praktischen klinischen Alltag zusammenzufassen.

**Zusatzmaterial online:**

Die Online-Version dieses Beitrags (10.1007/s00106-023-01280-3) enthält ein Manual für Atemtherapie bei Refluxbeschwerden.

## Abstract (en)

Laryngopharyngeal reflux (LPR) is characterized by backflow of gastric or gastroduodenal content and gases into the upper aerodigestive tract, which can damage the mucus membranes of the larynx and pharynx. It is associated with a variety of symptoms such as retrosternal burning and acid regurgitation, or other unspecific symptoms such as hoarseness, globus sensation, chronic cough, or mucus hypersecretion. Due to the lack of data and the heterogeneity of studies, diagnosis of LPR is problematic and challenging, as recently discussed. Moreover, the different therapeutic approaches are also discussed controversially in the face of the poor evidence base, and include pharmacologic and conservative dietary measures. Hence, in the following review, the available options for treatment of LPR are critically discussed and summarized for daily clinical use.

Der laryngopharyngeale Reflux (LPR) ist definiert als ein Aufstoßen von gastralem bzw. gastroduodenalem Sekret oder von gasförmigem Inhalt in den oberen aerodigestiven Trakt [24, 26]. Durch eine Schädigung der Schleimhäute u. a. im Larynx- und Pharynxbereich, aber auch durch Reizung von neuronalen Reflexbögen können dabei eine Reihe von Symptomen wie das klassische retrosternale Brennen und saure Aufstoßen getriggert und weitere andere teils sehr unspezifische Symptome wie Heiserkeit, „postnasal drip“, chronischer Husten, zervikales Globusgefühl oder Hypersekretion von Mukus im Larynx und Pharynx ausgelöst werden [2, 7, 45]. Aufgrund der heterogenen Studienlage und des Fehlens eines Goldstandards ist, wie kürzlich zusammengefasst, die Diagnosestellung des LPR schwierig und umstritten [44]: Dennoch kann die Diagnose eines LPR als wahrscheinlich betrachtet werden, wenn die Anamnese für ein Refluxgeschehen typisch ist, ggf. erhöhte Scores im Reflux Symptom Index (RSI) oder Reflux Symptom Score (RSS) vorliegen und keine Hinweise auf eine andere Grunderkrankung bestehen [3]. Eine apparative Diagnostik, die eine Endoskopie und eine oropharyngeale pH-Metrie einschließt, sollte aber angestrebt werden, da sich die LPR-Symptomatik so vielfältig und heterogen präsentieren kann [44]. Ist eine Diagnose gestellt, so kann eine mögliche Therapie des LPR konservative und medikamentöse Maßnahmen umfassen, welche jedoch zum Teil bei dünner und nicht konklusiver Datenlage umstritten sind [21]. Das Ziel der nachfolgenden Übersichtsarbeit ist daher, die verfügbaren therapeutischen Optionen des LPR zusammenzufassen und kritisch zu diskutieren.

## Medikamentöse therapeutische Möglichkeiten

### Protonpumpeninhibitoren

Die Therapie des LPR wird zwar grundsätzlich kontrovers diskutiert, dennoch hat sich das Behandlungsmanagement im Verlauf der letzten 20 Jahre kaum geändert. Sie basiert primär auf der Gabe von Protonenpumpeninhibitoren (PPI; Übersicht s. auch Tab. 1; [23]). Je nach Literatur beträgt der Anteil an Non-Responder-Patienten bis zu 40 %, welche nicht oder kaum auf diese empirische Therapie ansprechen [26, 30, 33]. Es ist ebenfalls nach wie vor unklar, ob der Einsatz von PPI im Vergleich zu Placebo tatsächlich zur signifikanten Abnahme der Symptomatik bei LPR führen kann [33, 41]. Die Komplexität der Problematik wird noch dadurch erhöht, dass es basierend auf neuen diagnostischen Verfahren nun auch eine Unterteilung in einen sauren, nichtsauren und gemischten LPR mit potenziell unterschiedlichem Ansprechen auf eine PPI-Therapie gibt [28]. Zudem kann das Refluat neben der Magensäure aus weiteren Komponenten wie Mikroorganismen, Pepsin und Gallensäuren zusammengesetzt sein, welche potenziell die ösophageale oder extraösophageale Schleimhaut schädigen können und allenfalls nur unzureichend durch eine PPI-Therapie beeinflusst werden.

**Table 1:** 

Gängigste Pharmaka	HWZ (h)	Zeit bis max. Plasmalevel (h)	Metabolismus	Kosten pro 14 St. (CHF; *EUR)	UAW
*Protonenpumpeninhibitoren*	–	–	–	–	Interaktionen: Hypochlorhydrie
Omeprazol 20, 40 mg	0,5–1	0,5–3,5	Hepatisch	15,45, 18,70 *6,06, *3,06	Kopfschmerzen, Diarrhö (abdominelle Beschwerden)
Esomeprazol 20, 40 mg	1–1,5	1,5	Hepatisch	14,65, 15,30 *11,56, *6,93	Kopfschmerzen, abdominelle Beschwerden
Lansoprazol 15, 30 mg	1,6	1,7	Hepatisch	9,80, 16,60 *6,78, *7,27	Kopfschmerzen, abdominelle Beschwerden
Dexlansoprazol 30, 60 mg	1–2	1–2, 4–5	Hepatisch	26,35, 28,85 *46,68, *51,11	Diarrhö, abdominelle Schmerzen, Übelkeit, abdominelles Unbehagen, Flatulenz, Obstipation, Kopfschmerzen
Pantoprazol 20, 40 mg	1–1,9	2–3	Hepatisch	8,63, 14,70 *2,46, *2,83	Diarrhö, Kopfschmerzen
Rabeprazol 20 mg	1–2	2–5	Hepatisch	20,50 *3,41	Kopfschmerzen, Diarrhö, Übelkeit
*H*_*2*_*-Rezeptorantagonisten* → aktuell in der Schweiz und großen Teilen von Europa nicht erhältlich	–	–	–	–	–
*Prokinetika*	–	–	–	–	–
Domperidon 10 mg	7–9	1–1,5	Hepatisch	3,45 *10,26	Kopfschmerzen, Diarrhö, Pruritus, Depressionen, Asthenie
Metoclopramid 10 mg	4–5	0,5–2	Hepatisch	2,21 *8,51	Kopfschmerzen, Diarrhö, Pruritus, Depressionen, Asthenie, extrapyramidale Effekte
*Alginate und Magaldrate* → rein direkte physikalische Wirkung	–	–	–	–	–
Alginat (Gaviscon®)	–	–	–	13,90 *5,38	Selten allergische Reaktionen
Magaldrat (Riopan®)	–	–	–	4,36 *6,27	Breiiger Stuhl

PPI werden seit rund 25 Jahren im klinischen Alltag eingesetzt, insbesondere zur Behandlung von säureassoziierten Erkrankungen wie dem gastroösophagealen Reflux oder peptischen Ulzerationen [49]. Ihren Wirkmechanismus entfalten PPI, indem sie die aktive Säuresekretion von Parietalzellen im Magen durch kovalente Bindung an die H^+^/K^+^-ATPase hemmen. PPI sind im sauren Magenmilieu allerdings labil, werden hier vorzeitig aktiviert und verlieren dadurch ihre Wirkung. Um dies zu verhindern, besitzen PPI-Tabletten eine magensaftresistente Beschichtung, durch die sie erst im Dünndarm aktiviert werden. Von dort können sie hämatogen in den Magen zurückzirkulieren, um dort ihre hemmende Wirkung auszuüben. Aufgrund der Aktivierung der PPI in saurem Milieu (pH ≤ 2,0–2,5) kann zudem eine hohe örtliche Selektivität und Sicherheit dieser Medikamente mit entsprechend geringer Anzahl unerwünschter Nebenwirkungen erzielt werden [47]. Pharmakologisch haben die klassischen PPI eine durchschnittliche Halbwertszeit von ca. 90 min. Eine Dosis von 20 mg hemmt dabei rund 70 % der Protonenpumpen, wodurch die Säuresekretion im Magen durchschnittlich über einen Zeitraum von ca. 24 h blockiert wird [48]. Ist eine maximale Einzeldosis im Normalfall von 40 mg erreicht, so kann die Inhibitionswirkung nicht durch eine weitere Dosiserhöhung, sondern nur durch die Erhöhung der Dosisfrequenz verbessert werden. Insgesamt kann so bei 12-stündlicher PPI-Gabe eine 80%ige Inhibition der Pumpleistung erreicht werden [35, 48]. Neben der säurehemmenden Wirkung der PPI kann über eine Erhöhung des pH-Werts im Magenlumen die Konversion von Pepsinogen zu Pepsin reduziert werden, sodass die Schleimhäute im Magen und Ösophagus, aber auch im laryngopharyngealen Bereich zusätzlich geschützt werden [20]. Diese Pharmaka haben allerdings keinen hemmenden Effekt auf die intrazelluläre Pepsinwirkung, das pankreatische Trypsin oder unkonjugierte Gallensäuren. So ist beispielsweise beschrieben, dass Trypsin synergistisch mit Gallensäuren zu einer ösophagealen und auch laryngopharyngealen Schleimhautschädigung führen kann [16]. Entsprechend wäre auch die laryngopharyngeale Schleimhaut im Rahmen eines LPR bei einem nichtsauren oder gemischten (duodenalen) Refluat nicht durch eine PPI-Gabe geschützt oder könnte im Fall des pankreatischen Trypsins durch die Erhöhung des pH-Werts bei PPI-Therapie sogar zusätzlich geschädigt werden [17, 18]. Ebenfalls sollte in diesem Zusammenhang angemerkt werden, dass durch die PPI-Therapie zwar die Säurekonzentration im Refluat gesenkt, nicht jedoch die Häufigkeit der täglichen ösophagealen und laryngopharyngealen Refluxepisoden selber reduziert wird [42, 52].

PPI gehören bei der Therapie des ösophagealen und laryngopharyngealen Refluxes zwar zu den am meisten rezeptierten Medikamenten, im Vergleich zu Placebo konnte jedoch allenfalls nur eine leichtgradige Wirkung der PPI in Bezug auf den LPR demonstriert werden [32, 33]. So konnte in einer Studie bei Patienten mit LPR durch eine PPI-Einnahme zwar eine verbesserte Symptomkontrolle der klassischen Refluxsymptome wie dem retrosternalen Brennen beschrieben werden. Chronische, extraösophageale und insbesondere laryngopharyngeale Beschwerden konnten demgegenüber durch die PPI-Therapie in einer anderen Studie nicht signifikant gebessert werden [51].

Weiterhin sind in jüngster Vergangenheit auch Zweifel über den Kurz- und Langzeiteffekt dieser PPI-Therapie aufgekommen, welche in einer kürzlich veröffentlichten Übersichtsarbeit zusammengefasst wurden (Tab. 1; [1]). Darunter sind auch seltene Störungen wie eine bakterielle Fehlbesiedlung des Dünndarms, Vitamin-B12-Mangel, eine Hypomagnesiämie oder Clostridium-difficile-Infektionen beschrieben [1]. Bei sehr schwachem Evidenzgrad wird zudem ein Zusammenhang der PPI-Einnahme mit Osteoporose, chronischer Niereninsuffizienz, Demenz, ambulant erworbenen Pneumonien oder kardiovaskulären Ereignissen kontrovers diskutiert [1].

Zusammenfassend kann auch im Hinblick auf die ungenügende Datenlage zur Therapie des LPR keine abschließende, konklusive Empfehlung bezüglich der PPI-Therapie abgeben werden. In Zusammenschau der dargelegten Fakten wird von zahlreichen Experten jedoch weiterhin eine probatorische PPI-Therapie (z. B. 40 mg Pantoprazol 2 × tgl.) für 2 Monate empfohlen. Eine zu kurze Einnahme von nur 3–4 Wochen, wie es im klinischen Alltag gehäuft verordnet wird, ist nicht zielführend. Vor Therapiebeginn könnte bei Unsicherheiten der Diagnose oder zur Abgrenzung eines sauren von einem nichtsauren Reflux noch eine ergänzende oropharyngeale oder ösophageale 24-h-pH-Metrie durchgeführt werden und bei der Therapieentscheidung hilfreich sein [44, 45].

### H_2_-Rezeptorantagonisten

Eine weitere Möglichkeit in der Hemmung der gastralen Säureproduktion besteht in der Applikation von H_2_-Rezeptorantagonisten. Bisher konnte in verschiedensten Studien kein Vorteil durch den Einsatz von H_2_-Rezeptorantagonisten im Vergleich zu einer PPI-Gabe bei säureassoziierten Erkrankungen wie dem gastroösophagealen Reflux, Magengeschwüren oder der Zollinger-Ellison Krankheit identifiziert werden. Im Hinblick auf die im vorherigen Kapitel zu den PPI beschriebene nächtlich vermehrte Magensäureproduktion konnte zwar durch die zusätzliche abendliche Gabe eines H_2_-Rezeptorantagonisten eine Reduktion der Säureproduktion erzielt werden. Dieser interessante Aspekt wurde bis anhin aber nicht in Bezug auf den LPR untersucht [46]. In verschiedenen Studien wurde untersucht, ob die gleichzeitige Gabe von H_2_-Rezeptorantagonisten mit einem PPI im Vergleich zu einer zweimal täglichen PPI-Gabe zu einer zusätzlichen Besserung führt. Bisher konnte jedoch kein klinischer Nutzen für die Anwendung in der Therapie der LPR abgeleitet werden. Zudem sind auch die Kosten einer Kombinationstherapie höher [4, 42, 50, 58].

### Prokinetika

Prokinetika fördern die Vorwärtsperistaltik und hemmen die Rückwärtsperistaltik der Darmmotilität und werden vor allem bei Übelkeit und Erbrechen oder bei Obstipation eingesetzt. Der Einsatz bei einer gastroösophagealen Refluxerkrankung (GERD) wird in der Literatur kontrovers diskutiert, und auch der Einsatz in der Behandlung des LPR wurde schon untersucht, mit gemischten Ergebnissen [12]. In einer randomisierten kontrollierten Studie konnte eine verbesserte Symptomkontrolle bei gleichzeitiger Gabe von Prokinetika in Kombination mit einem PPI nachgewiesen werden [8]. Diese Ergebnisse konnten in einer Studie von Hunchaisri et al. jedoch nicht bestätigt werden [15]. Dadurch könnte auch die Rolle des unteren Ösophagussphinkters (UÖS) in der Pathogenese des LPR hinterfragt werden, da der Sphinkterdruck des UÖS durch Prokinetika erhöht wird. Aktuell kann gemäß der aktuellen Studienlage keine Empfehlung bezüglich des Einsatzes von Prokinetika bei LPR abgegeben werden [12].

### Alginate und Magaldrate

Der Anwendungsbereich von Alginaten findet sich vor allem in der Medizin, aber auch der Nahrungsmittelindustrie. In der Medizin werden sie als Bestandteil von Verbandsmaterial eingesetzt oder als Kombinationspräparat mit Natriumbicarbonat und Calciumcarbonat zur symptomatischen Therapie von gastroösophagealem Reflux und Sodbrennen. Es bildet mit der Magensäure einen Gelschaum, welcher dem Mageninhalt aufliegt und so die Schleimhäute vor der Magensäure schützt. Zusammenfassend kann beispielsweise das Präparat Natriumalginat (Gaviscon®) einen schützenden Biofilm über die Schleimhäute des Magens, des Ösophagus sowie über den oberen Atemwegen formen, welcher über rund 4 h seine schützende Wirkung behält [55].

**Figure 1:**
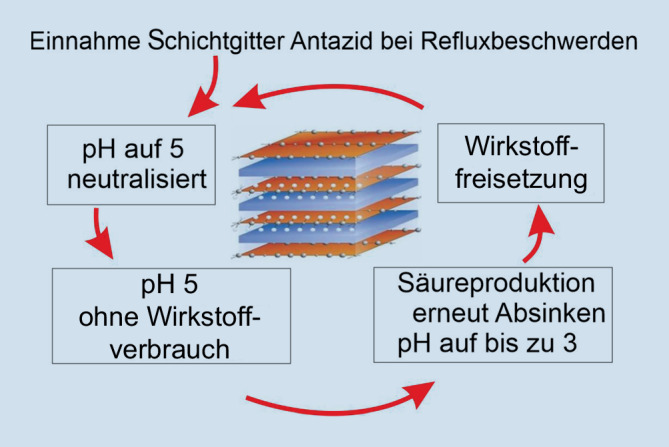

Magaldrate hingegen wirken antazid, indem sie als Schichtgitterantazid H^+^-Protonen binden und so den pH-Wert neutralisieren. Schichtgitterantazida neutralisieren mit ihrer speziellen Gitterstruktur nur so viel Magensäure, dass der optimale pH-Bereich von 3–5 aufrechterhalten bleibt (Abb. 1; [13]). So werden die Beschwerden effektiv durch das Präparat beseitigt, während die Magenfunktion weiterhin erhalten bleibt [13].

Vor allem für den nichtsauren oder gemischten Reflux könnte der Einsatz von Alginaten oder Magaldraten sinnvoll sein, da sie eine Art Schutzschicht über die Magen-, die Ösophagusschleimhaut und den oberen aerodigestiven Trakt bilden und somit auch vor nichtsauren gastroduodenalen Komponenten (Trypsine, konjugierte und nichtkonjugierte Gallensäuren) schützen [29, 34]. Zudem konnte interessanterweise gezeigt werden, dass dies im Gegensatz zu PPI die Häufigkeit von Refluxepisoden reduzieren kann [61]. Somit könnten Alginate als Alternative oder Ergänzung einer PPI-Therapie bei nichtsaurem oder gemischtem Reflux in Betracht gezogen werden [29, 54]. Daten hierzu und vor allem in Bezug auf den LPR sind hier jedoch noch ausstehend.

## Diätetische Maßnahmen

In Bezug auf die therapeutischen Möglichkeiten einer Refluxerkrankung rückt das wachsende Bewusstsein über die Nebenwirkungen und auch Kosten einer pharmakologischen PPI-Therapie zunehmend in den Vordergrund, sodass auch das alternative Management und die Modifikation von Lifestyle-Faktoren wie eine Ernährungsumstellung, Kontrolle des Körpergewichts oder der Verzicht auf Alkohol bzw. Kaffee an Stellenwert gewinnen [19, 26, 27, 59]:

Bislang ist der Einfluss von Ernährung und diätetischen Maßnahmen vor allem im Hinblick auf die klassische GERD in experimentellen und klinischen Studien untersucht worden [53, 59]. Beispielsweise konnte auf diagnostischer Ebene mittels ösophagealer Impedanz-pH-Metrie gezeigt werden, dass Mahlzeiten mit hohem Fettanteil bei Patienten mit Refluxerkrankung, aber auch bei gesunden Probanden zu einer erhöhten postprandialen Säureexposition des Ösophagus führen [10]. Auch nach einer vegetarischen Mahlzeit ergibt sich im Vergleich zu einer fettreichen Fleischmahlzeit eine signifikant verminderte postprandiale ösophageale Säureexposition [37]. Bezüglich der praktischen diätetischen Maßnahmen sind bei der klassischen GERD neben dem Verzicht auf fetthaltige Nahrungsmittel und Schokolade auch der Verzicht auf Süßgetränke oder Alkohol empfohlen, wobei die klinische Datenlage hier weiterhin dünn und oftmals nicht konklusiv ist [19, 38, 39, 43, 59].

Auch beim LPR wurden diätetische Interventionen und Lifestyle-Modifikationen als wirksame Alternative zur PPI-Therapie beschrieben: Beispielsweise konnte Koufman et al. in einer nichtkontrollierten Studie einen positiven Effekt auf die LPR-Symptomatik durch eine strikte säurearme Diät sowie eine Umstellung auf alkalines Wasser demonstrieren [22, 25]. Auch eine spezielle pflanzenbasierte mediterrane Diät mit konsequenter Nutzung von alkalinem Wasser (> pH 8) konnte bei Zalvan et al. in retrospektivem Studiendesign eine Reduktion des Reflux Symptom Index (RSI) erzielen, was mit einer regulären PPI-Therapie vergleichbar war [60]. Zwei weitere und prospektiv kontrollierte Studien von Nanda et al. und Chappity et al. konnten zudem zeigen, dass eine PPI-Therapie in Kombination mit entsprechenden diätetischen Maßnahmen wie der Vermeidung von fettreicher Nahrung sowie Lifestyle Modifikationen (Tab. 2) im Vergleich zur alleinigen PPI-Therapie mit einem überlegenen Therapieerfolg verbunden war [6, 40]. Ähnliche Ergebnisse haben sich auch in einer kürzlich erschienenen retrospektiven Studie gezeigt, bei der sich eine PPI-Therapie in Kombination mit diätetischen Maßnahmen und einer Lifestyle-Adaptierung der alleinigen PPI-Therapie überlegen gezeigt hat [31]. Bei den zuletzt genannten Studien ist eine Aussage über die isolierte Wirkung der diätetischen Interventionen selbst allerdings aufgrund des Studiendesigns nicht möglich.

**Table 2:** 

Lifestyle-Modifikationen bei GERD	
*Verzicht auf*	Heißes, stark gewürztes oder fettiges Essen
	Schokolade
	Alkohol und Nikotin
	Kaffee und Tee
	Schlafengehen innerhalb von 2 h nach dem Essen
	Stark zuckerhaltige Getränke
*Durchführung von*	Leichte Oberkörperhochlagerung bzw. Linksseitenlage beim Schlafen
	Vermeidung von starkem Räuspern
	Stimmschonung
	Gezielte Gewichtsabnahme/Erreichen von normalisiertem Körpergewicht

**Figure 2:**
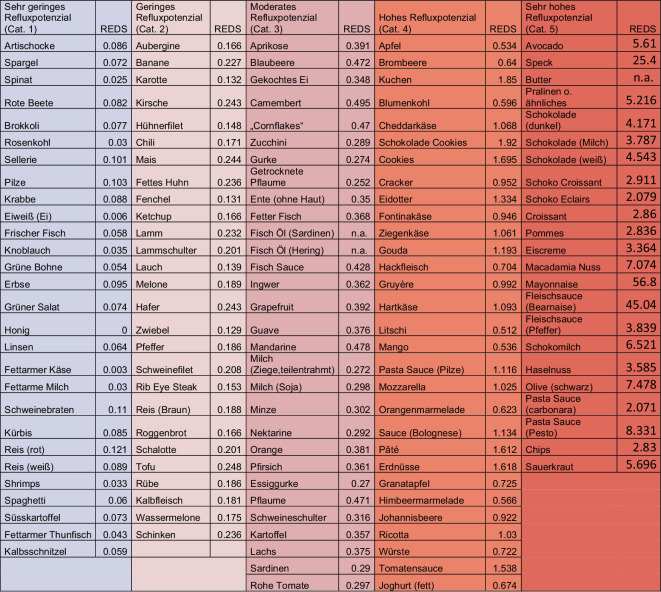

**Figure 3:**
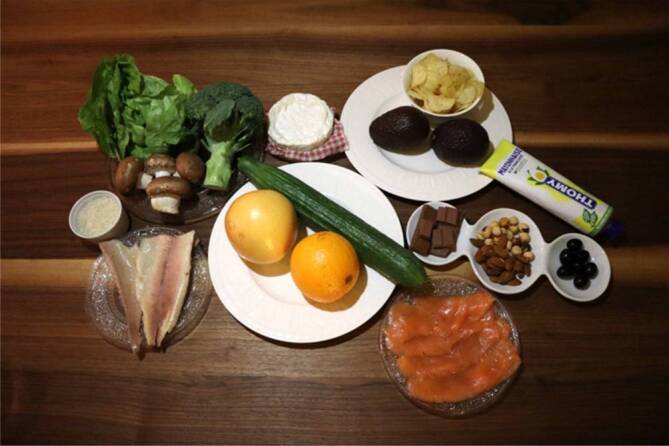

Ein neuer Ansatz zur Charakterisierung des Refluxpotenzials von diversen Speisen und Diäten wurde kürzlich von der LPR Study Group der Young Otolaryngologists initiiert [27]: Hierbei wurde der Versuch unternommen, nach Analyse von insgesamt 72 Studien und unter Berücksichtigung chemischer Gesichtspunkte wie beispielsweise pH-Wert, Lipid‑, Kohlenhydrat- und Faseranteilen, Alkoholkonzentrationen oder Osmolaritäten den Nahrungsmitteln ein direktes Refluxpotenzial, den sog. Refluxogenic Diet Score (REDS), zuzuordnen Abb. 2; [27, 32]. Dieser erlaubt eine Einteilung der diversen Nahrungsmittel in insgesamt 5 Kategorien von „very low reflux foods“ (Kat. 1) bis hin zu „very high reflux foods“ (Kat. 5; Abb. 2, Abb. 3; [27, 32]). Ebenso wurden diverse Getränke je nach refluxogenem Potenzial in verschiedene Klassen unterteilt, wobei hier insbesondere der pH-Wert und der Alkohol- bzw. Zuckeranteil Beachtung fanden (Tab. 3). Dabei ist die Klassifizierung der Getränke vom pH-Wert, glykämischen Index (Zuckeranteil mit Osmolarität), Kohlensäureanteil, Alkoholanteil und dem Vorhandensein von Koffein abhängig. Bei Vorhandensein von Kohlensäure, zusätzlichem Zucker oder einem Alkoholanteil von > 3 % erfolgt ein Upgrade der Kategorie (uCat).

**Table 3:** 

Getränk	pH-Wert	Cat	uCat
Alkohol (stark)	4	3	5
Aloe vera	6,1	2	2
Apfelsaft	3,65	4	5
Bier	4	3	5
Heiße Schokolade	6,3	2	3
Kaffee	5	3	4
Grapefruitsaft	3	4	5
Zitronensaft	2,3	4	5
Mehrfruchtsaft	3,8	4	5
Orangensaft	3,5	4	5
Soda (zuckerfrei)	2,5	4	5
Soda (mit Zucker)	2,5	4	5
Tee (Brombeere)	2,5	4	5
Tee (schwarz)	5,3	3	4
Tee (grün)	7	2	3
Tee (Zitrone)	2,9	4	5
Tomatensaft	4,35	3	3
Wasser (mit Kohlensäure)	7	2	3
Wasser (still)	7	2	2
Wasser (alkalisch)	8	1	1
Wein (rot)	4	4	5
Wein (rosé)	4	4	5
Wein (weiß)	4	4	5

*Kategorie 1 (Cat 1)*: „very low refluxogenic potential“, *Kategorie 2 (Cat 2)*: „low refluxogenic potential“, *Kategorie* *3 (Cat 3)*: „moderate refluxogenic potential“, *Kategorie 4 (Cat 4)*: „high refluxogenic potential“, *Kategorie 5 (Cat 5)*: „very high refluxogenic potential“

Je nach Menge der konsumierten Nahrungsmittelkomponenten kann dann wiederum ein Gesamtscore, der als sog. Refluxogenic Score of a Dish (RESDI) bezeichnet wird, zur Abschätzung des Refluxpotenzials einer vollen Mahlzeit errechnet werden [27, 32]. In diesem Zusammenhang lässt sich auch ein Global Refluxogenic Diet Score (GRES) ermitteln, mit dem das refluxogene Potenzial der Gesamternährung bzw. einer Diät abgeschätzt werden kann [27, 32]. Weitere Studien sind jedoch zur Validierung dieses neuen Systems erforderlich, bei denen die physiologischen Parameter wie beispielsweise die Säureexpositionszeit im oberen digestiven Trakt, aber auch die entsprechenden klinischen Verläufe nach Ernährungsumstellung charakterisiert werden sollten.

An dieser Stelle sollte auch erwähnt werden, dass zahlreiche Studien einen Zusammenhang zwischen der GERD und Übergewichtigkeit bzw. Adipositas belegen. So konnte beispielsweise demonstriert werden, dass gewichtsreduzierende Maßnahmen bei Adipositas mit einer deutlichen Verbesserung der ösophagealen Säureexpositionszeit wie auch der GERD-Symptomatik verbunden sind [11, 19].

Zusammenfassend kann auch bei aktuell noch unzureichender Datenlage angenommen werden, dass Ernährungsgewohnheiten bei der Pathogenese und Ausprägung der GERD wie auch dem LPR eine wesentliche Rolle spielen und entsprechende diätetischen Maßnahmen mit einem Benefit für die Patienten verbunden sein könnten. Unterstützend kann auch eine Gewichtsreduktion bei bestehendem Übergewicht sein.

## Logopädische und physiotherapeutische Therapieansätze

**Figure 4:**
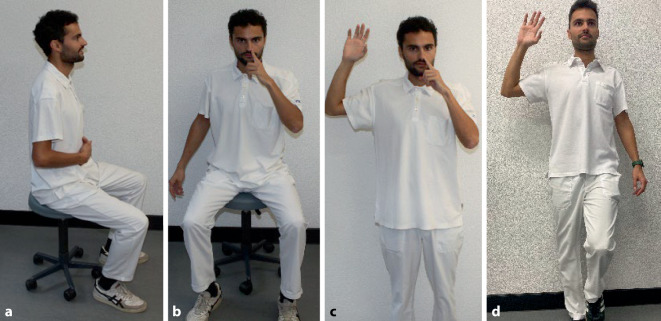

Neben den genannten Lifestyle-Maßnahmen werden weitere alternative Therapieformen bei Patienten mit GERD diskutiert, insbesondere auch der Effekt von logopädischen Maßnahmen [36]. Diese zielen einerseits darauf ab, die Verschlussfähigkeit vom UÖS zu verbessern. Dieser Sphinkter besteht u. a. aus den Crura des Diaphragmas, welche quergestreifte Muskulatur beinhalten und somit willkürlich beeinflusst werden können. Spezielle logopädische und physiotherapeutische Atmungsübungen sollen daher die Verschlussfähigkeit des UÖS verbessern können. Dies konnte bereits in einer kleinen Kohorte mittels manometrischer Verfahren demonstriert werden [5]. In einer anderen Studie wurden spezielle Atemübungen (Abb. 4) im Hinblick auf den gastroösophagealen Reflux untersucht, wobei ein 4‑wöchiges Training der Atemmuskulatur einen positiven Effekt auf den ösophagealen pH, die Lebensqualität und den Gebrauch von Protonenpumpeninhibitoren gezeigt hat [9]. Eine genauere Einschätzung des Stellenwerts der logopädischen und physiotherapeutischen Ansätze in der Therapie des LPR selber kann jedoch aufgrund der noch fehlenden Studien nicht abgegeben werden.

## Fazit für die Praxis

- Bei dünner Datenlage hinsichtlich der LPR-Behandlung bestehen bisher keine abschließenden und konklusiven Behandlungsrichtlinien.
- In Zusammenschau der verfügbaren Daten wird von zahlreichen Experten weiterhin eine probatorische PPI-Therapie für mindestens 2 Monate empfohlen, eine zu kurze Therapiedauer von 3–4 Wochen ist nicht zielführend.
- Im Fall eines fehlenden Therapieansprechens könnte alternativ zur oder kombiniert mit einer PPI-Therapie ein Alginat (z. B. Gaviscon®) eingesetzt werden, welches auch im Fall eines gemischten oder nichtsauren Refluxes einen positiven Effekt haben könnte.
- Diätetische Maßnahmen wie die Reduktion von refluxogenen Nahrungsmitteln und eine Verminderung von Übergewicht könnten ebenso wie physiotherapeutische Atemtherapien eine Alternative zur medikamentösen Therapie darstellen, wobei der Stellenwert bei diesen Therapien in Bezug auf den LPR noch ungenügend geklärt ist.

## Supplementary Information




