# Supplementary material for: Current possibilities and challenges in the treatment of laryngopharyngeal reflux
Source: HNO. 2023 Feb 16;71(5):294–303. [Article in German] doi: 10.1007/s00106-023-01280-3 (PMC10125953; doi:10.1007/s00106-023-01280-3)
Supplement: Supplementary file 1 [file 106_2023_1280_MOESM1_ESM.pdf]

Atemtraining zur Verbesserung von Refluxbeschwerden

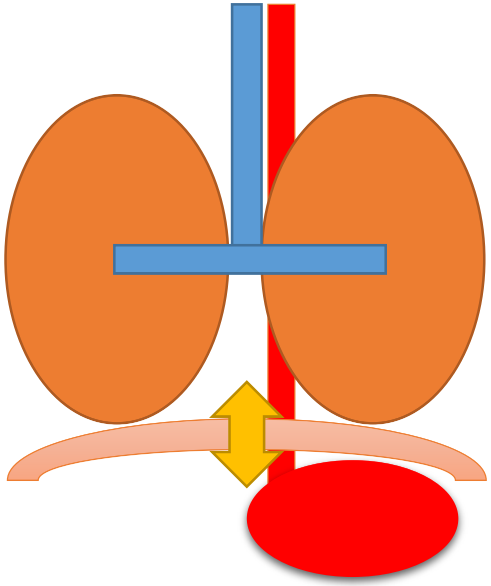

Vorwort:

Gemäss freundlicher Genehmigung durch Prof. Dr. med. Eherer konnten wir das Trianingsprogramm zur Verbesserung von Refluxbeschwerden modifizieren und unter Anderem auch für die klinische Verwendung bei uns einführen. Die Übungen stammen ursprünglich aus eienem Trainingsprogramm, das Prof. Karl Ernst Hoffmann (Gesangslehrer an der Musikhochschule Graz) mit seinen Schülern durchführt. Inhalte wurden zusätzlich aus einer Broschüre von Gernot Pusching übernommen und angepasst.

Allgemeine Hinweise

- Die Übungen nehmen ca. 20-30min Zeit in Anspruch.
- Probieren Sie sich während der Übungen zu entspannen um ein optimales Ergebnis zu erzielen.
- Wenn Sie sich während der Übungen unwohl fühlen oder Beschwerden bekommen brechen Sie die Übungen so lange ab und atmen Sie normal, bis sie wieder vollständig beschwerdefrei sind, um dann mit dem Training fortzufahren.
- Die Übungen sollten möglichst zur gleichen Tageszeit durchgeführt werden.
- Die Übungen sollten möglichst vor dem Essen oder min. eine Stunde nach dem Essen durchgeführt werden.
- Bei akuten Refluxbeschwerden können Sie sich auch auf eine Übung beschränken.
- In der ersten Woche sollten Sie min. 5x, in den folgenden Wochen 6-7x trainieren.
- Bitte dokumentieren Sie jedes Training und die eingenommene Medikation.

Ausgangspositionen

Liegend

- Legen Sie sich auf dem Rücken auf den Boden z.B. auf einer Decke oder Yogamatte.
- Stellen Sie die Beine auf.
- Vermeiden Sie ein Hohlkreuz der Lendenwirbelsäule.
- Lagern Sie ihren Kopf auf ein Polster/flaches Kissen.
- Legen Sie Ihre Arme locker neben den Körper.

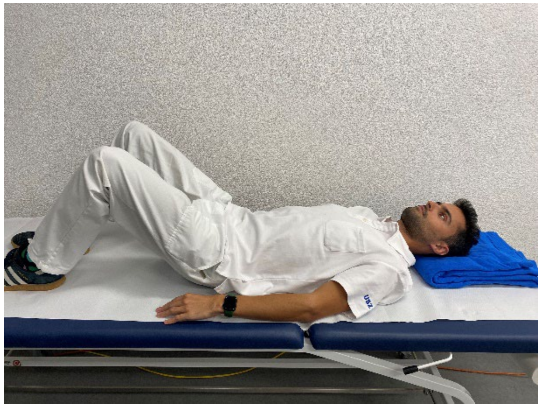

Sitzend

- Setzen Sie sich aufrecht auf einen Hocker/Stuhl.
- Die Knie sollten hüftbreit auseinander stehen.
- Die Füsse stehen flach auf dem Boden.
- Der Oberkörper wird aufgerichtet, das Becken sollte sich in Mittelstellung befinden.
- Heben Sie das Brustbein an und strecken Sie die Halswirbelsäule nach oben. (Als würden Sie am Hinterkopf nach oben gezogen werden, Kinn gen Kehlkopf)
- Lassen Sie die Schultern hängen und legen Sie Ihre Hände auf den Oberschenkeln ab.

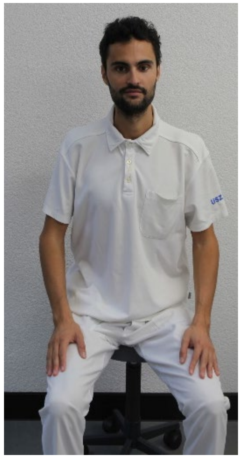

Ausgangsposition liegend

Übung 1

- Ausgangsstellung liegend.
- Legen Sie die linke Hand auf das Brustbein und die rechte Hand auf den Nabel.
- Atmen Sie tief durch die Nase ein, als würden sie einen angenehmen Duft einsaugen wollen.
- Achten Sie bei der Atmung auf die Bauchhebung, während der Brustkorb ruhig bleibt (Kontrolle des Bauches durch linke Hand, rechte Hand wird angehoben)
- Atmen Sie durch die Nase aus und Unterstützen Sie dabei das Einsinken des Bauches mit der rechten Hand.
- Wiederholen Sie die Übung 5-10x und tauschen Sie dann die Position der Hände

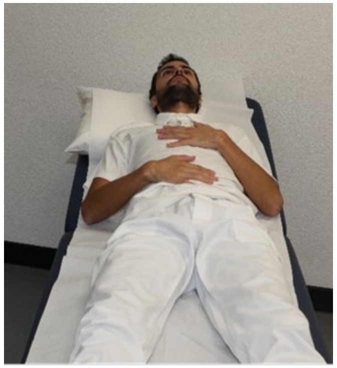

- *Steigerung: Legen Sie nach einer Woche ein 1kg schweres Gewicht auf den Bauch (z.B. Buch) und steigern Sie sich jede Woche um ein weiteren kg. Dokumentieren Sie das Gewicht im Protokoll.*

Übung 2

- Legen Sie beide Hände seitlich an den unteren Brustkorb.
- Atmen Sie tief durch die Nase ein, als würden sie einen angenehmen Duft einsaugen wollen.
- Heben Sie beim Einatmen zunächst den Bauch mit anschliessender Öffnung der unteren Rippen (Kontrolle des Brustkorbes durch die Hände).
- Achten Sie darauf, dass der obere Brustkorb sich nur wenig hebt.
- Atmen Sie durch die Nase aus.
- Starten Sie mit 5 Atemzügen und anschliessender Pause und steigern Sie im Verlauf auf 10 Atemzüge.

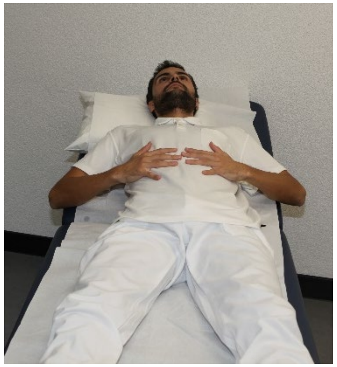

- *Steigerung: Erzeugen Sie bei der Einatmung einen Widerstand gegen das Öffnen des unteren Brustkorbes, jedoch nur so stark, dass noch eine gleichmässige Atmung möglich ist.*

Ausgangsposition sitzend

Übung 3

- Legen Sie beide Hände übereinander auf den Nabel.
- Atmen Sie ruckartig/schnuppernd durch die Nase ein (anfangs ca. 5 Impulse),während Sie leichten Druck mit den Händen auf den Bauch ausüben bis ein angenehmes Spannungsgefühl entsteht.
- Halten Sie diese Spannung ca. 3 Sekungen.
- Atmen sie langsam (über ca. 10 Sekunden) über gespitze Lippen aus.
- Führen Sie anfangs max. 5 Wiederholungen aus und machen Sie zwischendurch Pausen mit 2-3 normalen Atemzügen.

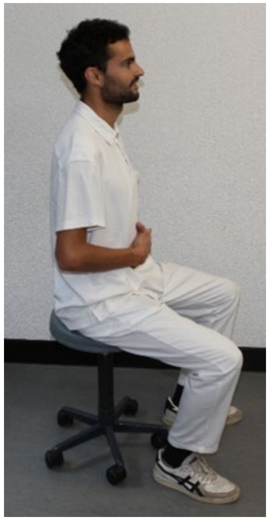

- *Steigerung: Erhöhen Sie innerhalb der 2.-4. Woche*
    - *die Impulse langsam auf 10*
    - *das Halten der Spannung auf 10 Sekunden*
    - *sowie das Ausatmen auf 20 Sekungen.*

Übung 4

- Atmen Sie tief durch die Nase ein, als würden sie einen angenehmen Duft einsaugen wollen und heben Sie dabei den rechten Arm seitlich am Kopf nach oben.
- Atmen Sie langsam und ruhig durch die Nase aus und senken Sie dabei den Arm.
- Wiederholen Sie die Übung zunächst 5x rechts und dann 5x links.
- Die jeweils freie Hand kontrolliert den Bauch.

Ausgangsposition stehend

Übung 4

- *Steigerung:*
- *Woche: Halten Sie jeweils mit der freien Hand ein Nasenloch zu.*
- *3. Woche: Ausgangsstellung aus dem Stand. Halten Sie jeweils ein Nasenloch zu und heben und senken Sie die freie Hand bei jedem Atemzug.*
- *4. Woche: Ausgangsstellung aus dem Einbeinstand. Rechter Arm hebt mit linkem Bein und linker Arm mit rechtem Bein.*

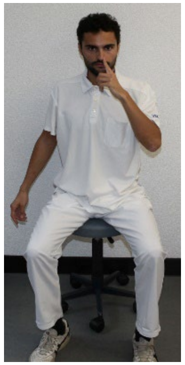

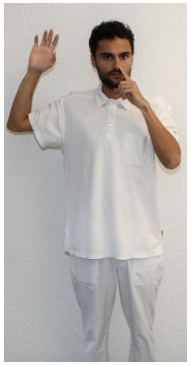

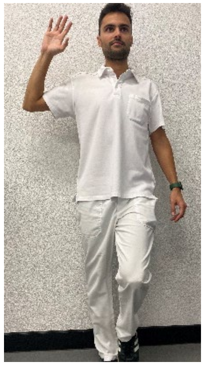

Übung 5

- Atmen Sie ruckartig/schnuppernd ein.
- Halten Sie beim Einatmen die Atemspannung.
- Setzten sie mit den Vokale /o/, /a/, und /e/ stossartige Lachakzente gegen die Atemspannung (lautstärke mittel bis stark, angenehme Tonhöhe). Dabei möglichst keine Luft ausstossen (nicht ho-ho-ho, sondern o-o-o)
- Beginnen Sie mit 8-10 Lachimpulsen.
- Wiederholen Sie die Übung nach einigen Atemzügen mit Luftstoss (nun ho-ho-ho)
- Steigerung
  - Steigerung auf bis zu 25 Lachimpulse.
  - Abwechseln (ja Atemzug) mit und ohne Luftstoss (ho-o-ho-o) üben
